# Factors Influencing Individuals’ Hesitation to Provide First Aid in Emergencies

**DOI:** 10.7759/cureus.98214

**Published:** 2025-12-01

**Authors:** Suliman Hadadi, Nawaf Alnuwaysir, Talal Almadani, Reem Alsaeed, Ruqayyah Hadadi, Bayader Alotaiby, Faisal Alotaibi, Mohammed Almadhi, Lama Alshehri

**Affiliations:** 1 Department of Community Health Sciences, King Saud University, Riyadh, SAU; 2 College of Health and Rehabilitation Sciences, Princess Nourah bint Abdulrahman University, Riyadh, SAU; 3 Directorate of Studies and Change Management, Ministry of Health, Riyadh, SAU

**Keywords:** bystander, emergency response, first aid, hesitation, riyadh, saudi arabia

## Abstract

First aid is a life-saving skill, yet many bystanders hesitate to intervene during emergencies due to various psychological, social, and consequential barriers. This cross-sectional study investigated the factors influencing first aid hesitancy among residents of Riyadh, Saudi Arabia. A total of 1,124 participants aged 18 and above completed an online questionnaire assessing their demographic profiles, first aid knowledge, experience, and perceived barriers. Results showed that 53% (n= 596) had received first aid training, but only 20% (n= 225) held a formal license. While 71.2% (n= 795) reported low psychological barriers, over half faced moderate consequential barriers, such as fear of legal consequences or causing harm. Key barriers included fear of infection (73%; n= 815), discomfort with public attention (72.6%; n= 810), and hesitation in assisting the opposite sex (45.8%; n= 511). Despite these concerns, 93% (n = 1045) of respondents supported integrating first aid education into schools and workplaces. The findings highlight a gap between awareness and action, emphasizing the need for certified training and public knowledge education to empower individuals to act confidently during emergencies. Addressing these barriers can significantly enhance community preparedness and potentially save lives.

## Introduction

First aid is a critical skill that enables individuals to provide immediate care during medical emergencies before professional help arrives. Among first aid interventions, cardiopulmonary resuscitation (CPR) plays a vital role in saving lives during cardiac and respiratory emergencies. Accidents and emergencies can happen at any time, and knowing how to intervene can make a significant difference in preventing harm or death. For instance, in cases of cardiac arrest, immediate CPR can double or triple survival rates [1]. Unintentional injuries, however, remain a significant public health issue. In the United States, they were the leading cause of death among children aged 1 to 14 [2]. In Saudi Arabia, falls (31.9%) and motor vehicle accidents (25.1%) were among the top causes of childhood injuries [3].  

The bystander effect is described as failing in providing help for people in need due to others being on site [4]. Bystanders might hesitate to help due to fear, lack of knowledge or discomfort with assisting others. Adequate knowledge of when and how to perform first aid is essential, as insufficient understanding can increase the risk of causing unintentional harm to the affected individual. For instance, a recent study by Sepahvand et al. [5] found that bystanders’ fears and concerns are from a lack of knowledge about providing first aid. Moreover, a recent study by Jaskiewicz et al. [6] found that the most commonly reported barriers to performing CPR were fear of causing harm, disease transmission and uncertainty in identifying cardiac arrest. In Saudi Arabia, however, Barnawi et al. [7] reported that the most common barrier to performing CPR among the general population was the fear of causing harm, cited by 73.6% of participants. Alhussein et al. [8] examined the knowledge of non-healthcare individuals in Riyadh and found that 51.8% of participants were unfamiliar with CPR, while only 4.4% had received formal CPR training.  

Saudi Arabia has been working to increase first aid awareness among its population. Several national initiatives are being launched to increase the knowledge and practice of first aid and basic life support (BLS). These efforts aim to develop educational materials and programs, such as free courses, online booklets, and videos [9,10]. A notable initiative is the inclusion of first aid courses in secondary schools, which will be accessible to all students, with a program set to begin in 2025-2026 [11]. Increasing awareness and providing training can and might reduce these barriers, also empowering individuals to act confidently during emergencies. Despite having the opportunity to assist, many individuals hesitate or refrain from providing first aid during emergencies. Therefore, this study aims to identify and analyze psychological, social, and consequential factors influencing bystanders’ hesitation to provide first aid in Riyadh, Saudi Arabia. Understanding these indicators is essential for developing and/or suggesting strategies to improve public responsiveness and enhance emergency preparedness. 

## Materials and methods

Study design, study population, and sampling 

This study employed a cross-sectional study using a convenience sampling method, a type of non-probability sampling. Convenience sampling was chosen due to its feasibility and the ease of accessing a wide range of individuals across Riyadh city. The target population consisted of residents of Saudi Arabia, Riyadh aged 18 years and above. A total of 1,215 participants completed the survey. Data were collected using a self-administered online questionnaire previously evaluated for clarity, content validity, and internal consistency.

Data collection and survey instrument**

Data was collected through a self-administered online questionnaire distributed via popular social media platforms, including WhatsApp, LinkedIn, and X (formerly Twitter). Google Forms was used to collect the questionnaire, and the data collection period spanned from November 2023 to January 2024. 

The questionnaire was developed through five key phases. First, we organized the topics by grouping the factors that influence first aid behavior into psychological, social, legal, cultural, and situational categories. Next, we drafted the main questions to match each of these categories and capture their potential impact. We then included background information such as age, gender, and personal views to better understand the characteristics of the participants. After that, the questions were arranged in a clear and logical order to make the survey easy to follow. Finally, a thorough review was conducted to ensure that the questionnaire was clear, ethically appropriate, and ready for distribution.

The survey instrument consisted of four main sections. The first section focused on sociodemographic characteristics and collected information such as gender, age group, area of residence, employment status, and healthcare background. The second section explored participants’ experience and practice of first aid, including whether they had received formal training, possessed a first aid license, or had any prior experience providing assistance during emergencies. The third section addressed psychological, social, and legal barriers by assessing factors such as fear, anxiety, discomfort with helping strangers, and concerns about legal consequences that may hinder individuals from offering first aid. The fourth section covered situational and environmental challenges, examining external obstacles such as limited access to training, unclear regulations, environmental conditions, and the influence of social media during emergency situations. All items were measured using a 3-point Likert scale (Agree, Neutral, Disagree).

Validity and reliability**

Content validity of the questionnaire was confirmed through expert review by specialists in the public health field. A pilot test with 25 participants assessed clarity and item relevance, and minor modifications were made accordingly. Internal consistency reliability was evaluated using Cronbach’s alpha (0.85), indicating strong internal consistency.

Inclusion and exclusion criteria

Participants were included if they were 18 years of age or older and resided in Riyadh at the time of the study. A total of 1,215 responses were collected. However, 91 participants who reported living outside Riyadh were excluded from the analysis. After applying the exclusion criteria, the final sample consisted of 1,124 participants. 

Data analysis**

Data were analyzed using both JMP and Microsoft Excel. Descriptive statistics, including frequencies and percentages, were used to summarize categorical variables such as demographic characteristics, experience in first aid and healthcare, certifications, and levels of agreement. For the barrier analysis, each section, Psychological Barriers (Section 3), Social Barriers (Section 4), and Consequential Barriers (Section 5) included eight items measured on a 3-point Likert scale (Agree, Neutral, Disagree). Two items were reverse-coded prior to analysis: Q3 (“I am confident in my ability to give first aid”) in the psychological barriers section, and Q6 (“Giving first aid is rewarding and valuable”) in the consequential barriers section. Barrier levels were calculated and then categorized as follows: Low perceived barrier, moderate barrier, and high perceived barrier. Results were presented in tables and figures for clarity. 

Ethical considerations

Ethical approval was obtained from the Institutional Review Board (IRB) at King Saud University (Approval Number 25-634). The study followed the principles outlined in the Declaration of Helsinki. Secure data storage measures were applied to ensure that all collected responses were protected and accessible only to the research team. Before providing digital consent, participants were shown a brief explanation of the study’s purpose and their voluntary participation rights including that no identifying information would be collected and all responses would remain anonymous. Participation was voluntary, and informed consent was obtained electronically before starting the survey. Responses were anonymous, and participants were assured of confidentiality and the right to withdraw at any stage without any consequences. 

## Results

A total of 1,124 participants were included in the study. As shown in Table 1, the majority were female (n = 688; 61.21%), while male participants accounted for 38.79% (n = 436). The largest age group was 21-25 years old (n = 347; 30.87%), followed by 18-20 years old (n = 217; 19.31%). The most common occupational status was student (n = 472; 41.99%), followed by employed individuals (n = 390; 34.70%). In terms of geographic distribution, most respondents resided in the North of Riyadh (n = 402; 35.77%) and the East (n = 356; 31.67%). 57.8% (n= 645) of participants reported having a background in the health field in either work or knowledge, while 42.2% (n= 471) did not. 53.05% (n= 592) had undergone first aid training, and only 19.44% (n= 217) reported having a first aid license. 52.06% (n= 581) of respondents had never been involved in providing first aid during a crisis, while 26.52% (n= 296) reported some level of involvement.  

**Table 1:** Sociodemographic Characteristics of All Participants.

Sociodemographic characteristic	N	%
Gender		
Female	688	61.21%
Male	436	38.79%
Age		
18-20 Years old	217	19.31%
21-25 Years old	347	30.87%
26-30 Years old	131	11.65%
31-35 Years old	97	8.63%
36-40 Years old	119	10.59%
41-45 Years old	83	7.38%
46-50 Years old	55	4.89%
51-55 Years old	41	3.65%
56-60 Years old	34	3.02%
Occupational status		
Student	472	41.99%
Employed	390	34.70%
Employed and a student	114	10.14%
Unemployed	148	13.17%
Region of Riyadh		
North of Riyadh	402	35.77%
South of Riyadh	106	9.43%
West of Riyadh	194	17.26%
East of Riyadh	356	31.67%
Central Riyadh	66	5.87%
Experience/knowledge in healthcare		
Reported knowledge in the health field	441	39.52%
Reported work experience in the health field	204	18.28%
Reported no knowledge or experience in the health field	471	42.20%
First aid course certificate/licensing		
Has a first aid certificate	592	53.05%
Does not have a first aid certificate	524	46.95%
Has a first aid license	217	19.44%
Does not have a first aid license	899	80.56%
Experience with providing first aid		
Has not provided first aid in any crisis faced	154	13.80%
Provided first aid in some crises faced	296	26.52%
Provided first aid in most crises faced	85	7.62%
Has not faced or witnessed any crisis	581	52.06%

**Figure 1:**
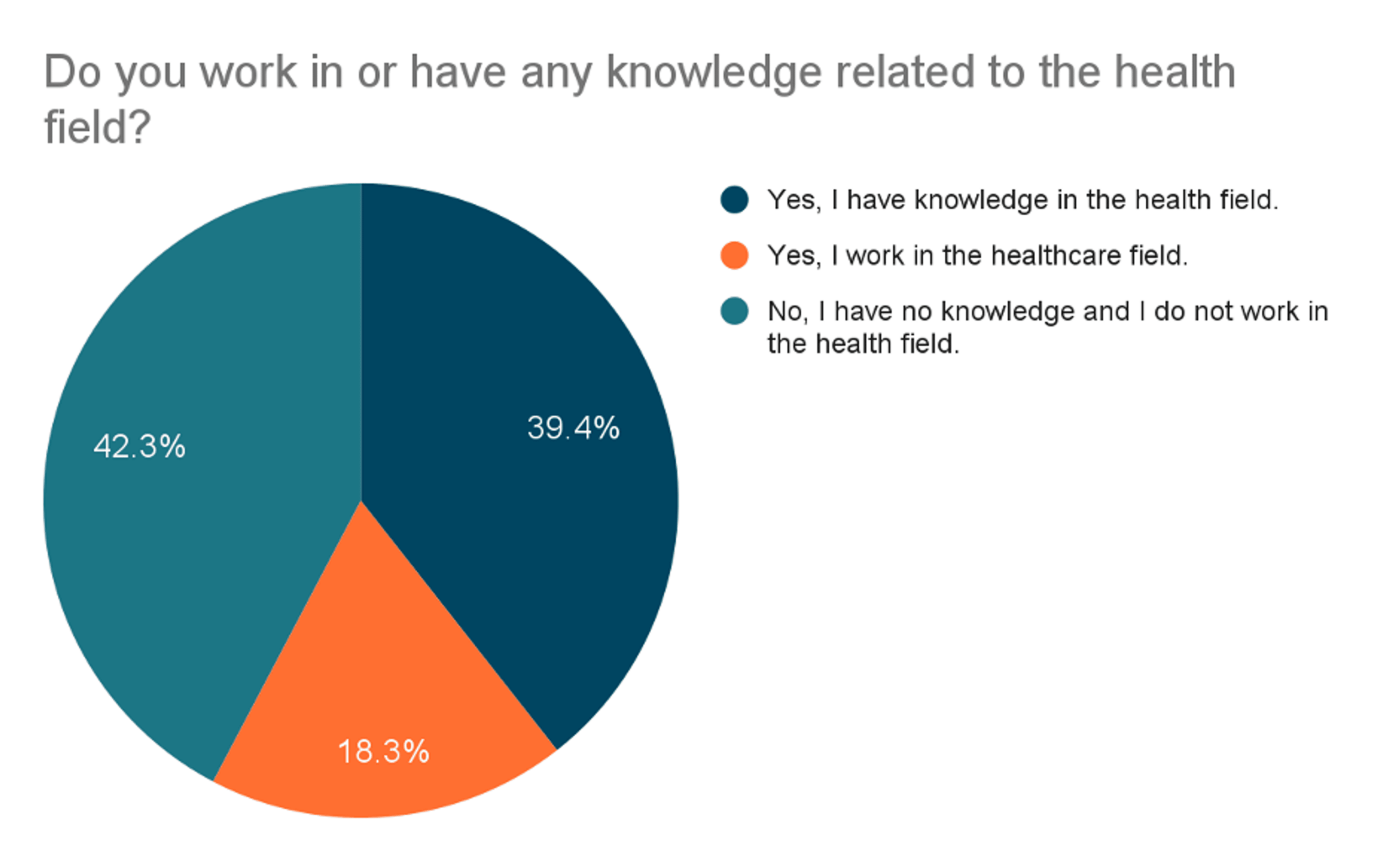
Respondents’ Background in the Health Field

As shown in Figure 1, 39.4% (n= 443) of participants reported having knowledge in the health field, while 42.3% (n= 474) did not.

**Figure 2:**
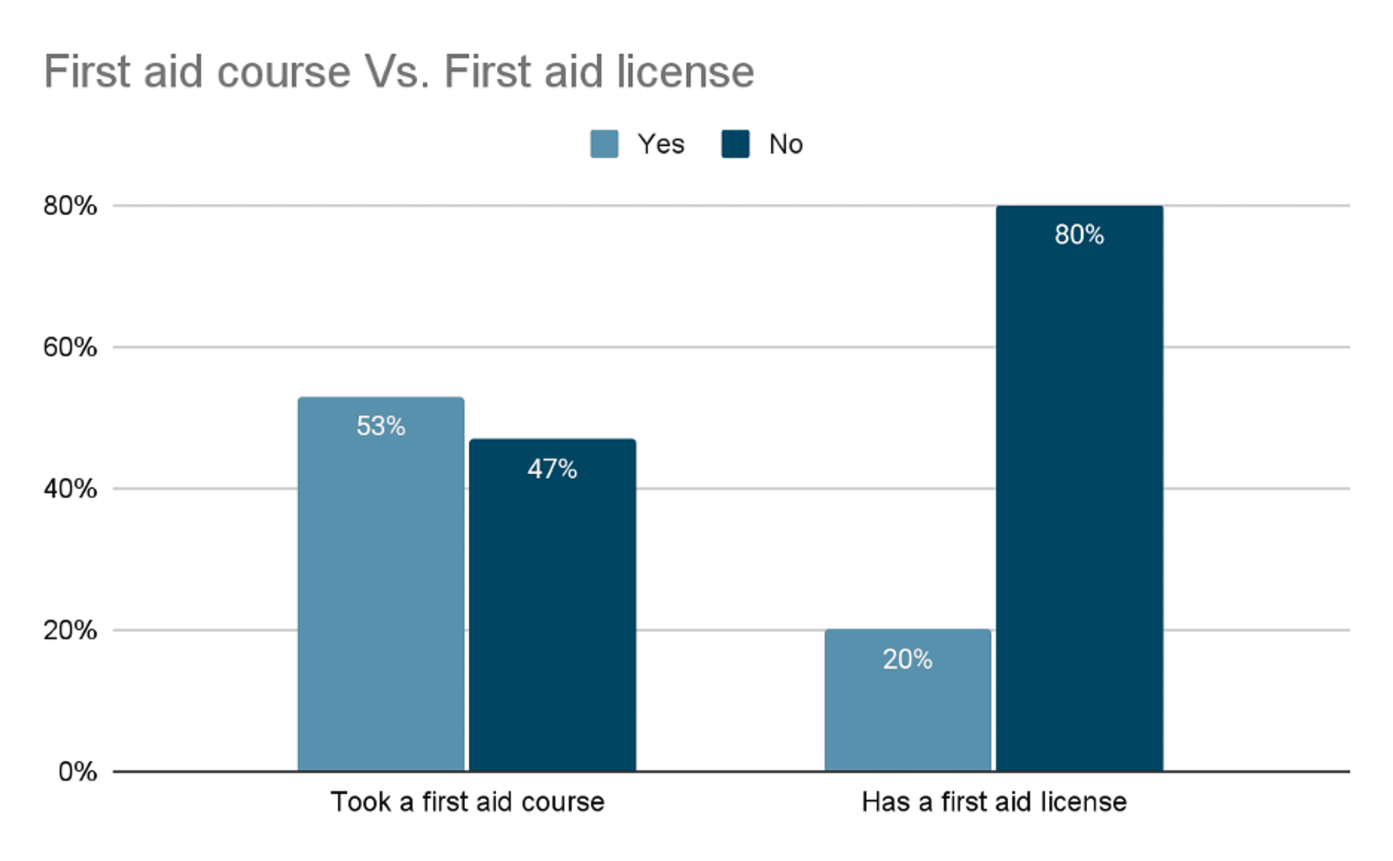
Comparison of First Aid Training and Licensing Among Respondents

In Figure 2, 53% (n= 596) had undergone a first aid course, and only 20% (n= 225) reported having a first aid license, compared to 47% (n= 528) without any course. 

**Figure 3:**
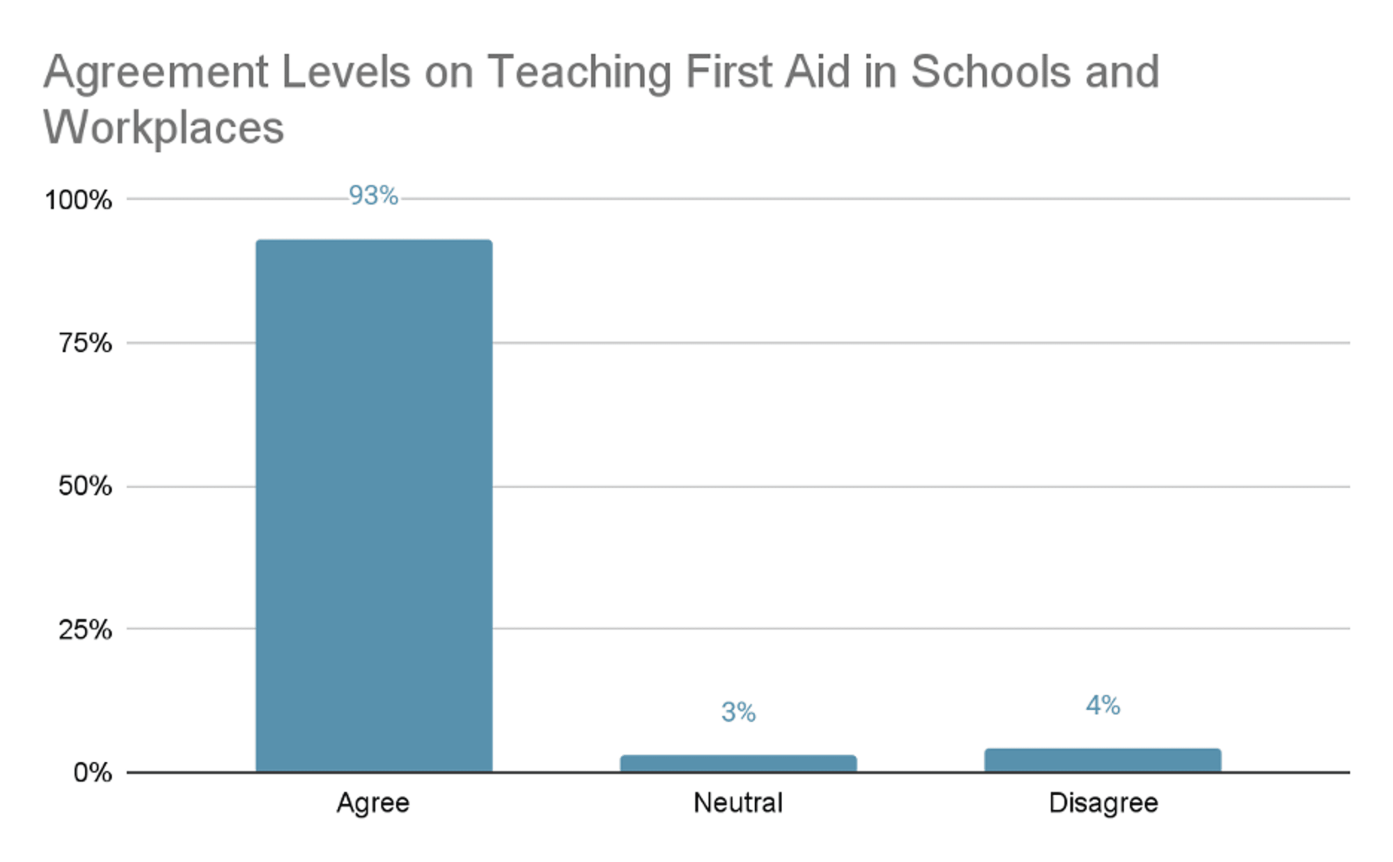
Perceptions of the Importance of First Aid Education in Schools and Workplaces

As shown in Figure 3, a majority of respondents, 93% (n= 1045), agreed that teaching first aid in schools and workplaces is important, while 3% (n= 34) were neutral and 4% (n= 45) disagreed, indicating strong overall support for first aid education.

**Figure 4:**
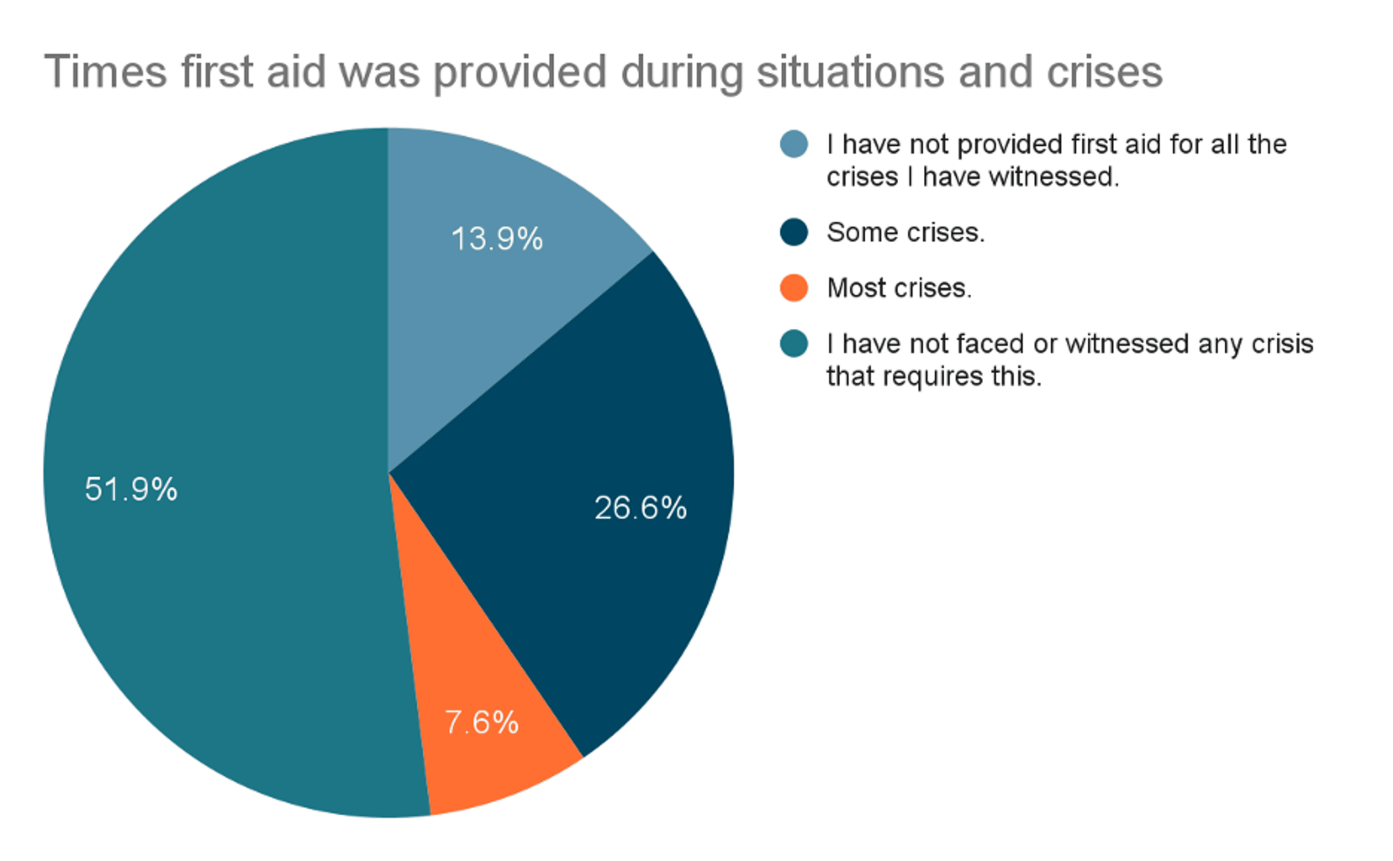
First Aid Involvement in Crisis Situations

According to Figure 4, 51.9% (n= 583) of respondents had never been involved in providing first aid during a crisis, while 34.2% (n = 384) reported at least some level of involvement (i.e., involvement in either some or most crises faced).

**Figure 5:**
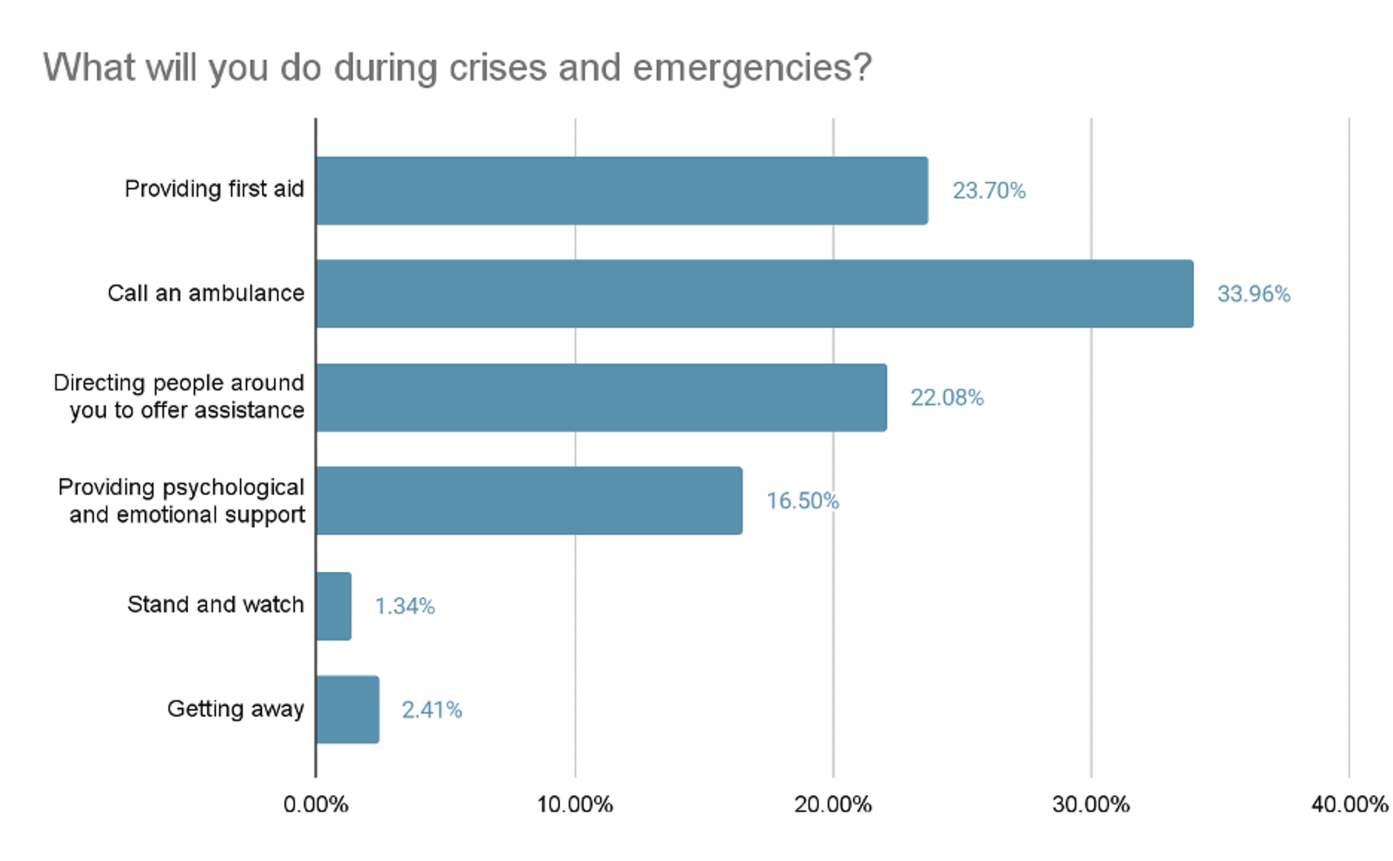
Intended Actions During Crises and Emergencies

In Figure 5, the most frequently selected intended action during emergencies was calling for help or emergency services (33.96%, (n= 382), whereas only 23.70% (n= 266) indicated that they would personally provide first aid. 

As shown in Table 2, the most commonly agreed-upon psychological barrier was having health issues preventing the application of first aid (87.19%; n = 973). For social barriers, 70.16% (n= 789) were discouraged by social pressure. Among consequential barriers, 56% (n= 629) believed first aid was difficult to learn. 

**Table 2:** Respondents’ Agreement with Barriers to First Aid Assistance

Section/Question	Agree		Neutral		Disagree	
	N	%	N	%	N	%
Psychological Barriers						
Psychological fear prevents me from providing first aid.	478	42.83%	434	38.89%	204	18.28%
Stress prevents me from providing first aid.	473	42.38%	411	36.83%	232	20.79%
The possibility of causing harm to the injured person prevents me from providing first aid.	249	22.31%	432	38.71%	435	38.98%
My fear of the injured person's reaction prevents me from providing first aid.	613	54.93%	310	27.78%	193	17.29%
Previous bad situations prevent me from providing first aid.	816	73.12%	207	18.55%	93	8.33%
I don't like to be the center of attention, so I don't give first aid.	810	72.58%	199	17.8 3%	107	9.59%
Fear of infection prevents me from providing first aid.	815	73.03%	208	18.64%	93	8.33%
I have health problems that prevent me from providing first aid.	973	87.19%	99	8.87%	44	3.94%
Social Barriers						
I feel uncomfortable providing first aid to people of the opposite sex.	511	45.79%	269	24.10%	336	30.11%
I'm worried about the legal consequences, so I don't provide first aid.	548	49.10%	355	31.81%	213	19.09%
I cannot predict the reaction of the people around the injured person, so I cannot provide first aid.	633	56.72%	328	29.39%	155	13.89%
I feel uncomfortable giving first aid to strangers.	683	61.20%	265	23.75%	168	15.05%
Society's lack of understanding of the importance of first aid prevents me from providing it.	709	63.53%	251	22.49%	156	13.98%
I don't prefer to help when I'm in another community.	650	58.24%	279	25.00%	187	16.76%
My lack of knowledge of the laws regarding first aid prevents me from providing it.	368	32.97%	348	31.18%	400	35.84%
Social pressures prevent me from providing first aid.	783	70.16%	242	21.68%	91	8.15%
Consequential Barriers						
Difficulty accessing first aid learning resources prevents me from providing them.	625	56.00%	273	24.46%	218	19.53%
Not having a first aid license prevents me from providing it.	392	35.13%	255	22.85%	469	42.03%
I feel that providing first aid is difficult and a big challenge.	376	33.69%	412	36.92%	328	29.39%
I rely on the people around the injured person, so I do not provide first aid.	459	41.13%	427	38.26%	230	20.61%
Not being sure that the person needs help prevents me from providing first aid.	443	39.70%	431	38.62%	242	21.68%
I believe that providing first aid is the job of paramedics and specialists, so I do not provide first aid.	599	53.67%	300	26.88%	217	19.44%
The environmental and climatic conditions surrounding me prevent me from providing first aid (rainy or stormy weather) or (the injured person is in a place that is difficult to reach, such as a public road)	527	47.22%	330	29.57%	259	23.21%
Social media prevents me from providing first aid (such as someone around me taking pictures or my unwillingness to contribute to an event that might be circulated on social media platforms)	570	51.08%	264	23.66%	282	25.27%

In Table 3, the majority of participants reported low psychological barriers (n = 795; 71.24%). Low social barriers were reported by 56.36% (n = 629), while moderate consequential barriers were the most prevalent in their category, affecting 53.14% (n = 593) of respondents. High consequential barriers were noted by 13.98% (n = 156) of participants. 

**Table 3:** Categorization of Barriers to Providing First Aid by Intensity Among Respondents

Variables	N	%
Psychological Barriers		
Low perceived psychological barrier	795	71.24%
Moderate psychological barrier	285	25.54%
High perceived psychological barrier	36	3.23%
Social Barriers		
Low perceived social barrier	629	56.36%
Moderate social barrier	378	33.87%
High perceived social barrier	109	9.77%
Consequential Barriers		
Low perceived consequential barrier	367	32.89%
Moderate consequential barrier	593	53.14%
High perceived consequential barrier	156	13.98%

## Discussion

To the best of the available knowledge, this is one of the first studies to comprehensively examine a wide range of barriers to providing first aid, capturing diverse perspectives from citizens across Riyadh city. This study identifies key barriers to providing first aid in emergencies and offers valuable insights into the perceptions of Riyadh citizens, highlighting the need for further investigation. The majority of respondents were female (61.2%; n= 688) and within the 21-25 age group (30.9%; n= 347), with students comprising the largest group 42% (n= 472). Around 57.7% (n= 645) of participants had a health-related background, either through knowledge or work. The World Health Organization (WHO) supports engaging communities by equipping them with the essential knowledge and skills in first aid through training-of-trainers programs [12]. Certification plays a crucial role in giving individuals the confidence to provide first aid and might reduce hesitation during serious emergencies. Table 1 shows that while 53% (n= 592) had received first aid training, only 19.4% (n= 217) have a formal first aid license. Similar gaps were identified in a recent study by Bashekah et al. [13], who found moderate mean knowledge, yet only one-third have received training in first aid. Similarly, some medical students who had not received first aid training demonstrated low levels of knowledge [14]. These findings stress the importance of offering accessible, certified training programs for everyone, especially those in the health field.  

Although our study identifies some knowledge barriers and confidence gaps, other global studies suggest additional barriers to first aid course attendance include cost and limited accessibility. Yin et al. [15] found that community health workers in China identified limited resources, lack of professional training, and insufficient support as major barriers to delivering first aid training. Similarly, Pandey et al. [16] reported that financial and low-level knowledge of first aid were barriers in Nepal. These findings suggest that many participants reported significant psychological and social barriers, indicating a need for further research to explore these factors in depth.

Most participants felt that providing first aid is difficult and a major challenge, with 33.7% (n= 376) agreeing, 36.9% (n= 412) remaining neutral, and 29.4% (n= 328) disagreeing, raising concerns about confidence and readiness in emergencies. Offering first aid courses in schools and workplaces may better equip the community with essential knowledge, which may help reduce hesitation and improve responsiveness. Moreover, 56% (n= 625) of participants reported that difficulty accessing first aid learning resources prevented them from providing aid. In Saudi Arabia, some organizations and initiatives offer certified first aid and BLS training courses for a fee, yet some individuals may struggle to afford the associated costs or may not be aware that such training opportunities exist. Thus, this study highlights strong public support for integrating first aid education/courses into schools and workplaces, with 93% (n = 1045) of respondents agreeing on its importance. A systematic review in Saudi Arabia found that many teachers do not know enough about first aid, which can affect how well they respond in emergencies [17]. In terms of demands, a study found that 47.2% supported mandatory CPR training in schools, and 31.3% supported it in every job [18]. The widespread agreement in our sample and other papers suggest that the community is open and receptive to structured first aid programs. These findings highlight the need to integrate certified first aid programs into academic institutions and workplaces.  

Table 3 shows that most participants 71.24% (n=795) reported low psychological barriers, suggesting general confidence in providing first aid. However, only 56.36% (n= 629) reported low social barriers, indicating some concern about social judgment. Notably, 53.14% (n= 593)** **faced moderate consequential barriers, such as fear of legal issues or harming the victim. These findings indicate that while many feel mentally prepared, external and outcome-related concerns may still hinder first aid response. When asked how they would respond during a crisis, nearly 34% (n= 382) of participants said they would call an ambulance, 23.7% (n= 266) would provide first aid, while only 1.34% (n=15) chose to act as a bystander. Diving into the barriers, (n= 633) 56.7% indicated that they could not predict how others would react, which made them hesitant to provide first aid. Additionally, (n= 459) 41.1% reported that they relied on others at the scene to perform first aid. A study by Abdelrahman et al. [19] conducted among female school educators in Riyadh found that 974 participants (92%) believed first aid should be administered not only by medical professionals but also by bystanders during emergencies. In contrast, in this study, over half of participants (n=599) agreed that providing first aid is solely the responsibility of healthcare professionals. As a result, people may be more likely to remain on the bystander instead of offering help and potentially saving lives. This also contrasts with findings from a Jordanian study [20] where 71.6% of participants (n=518) believed that helping accident victims should not be left to healthcare workers alone. 

In this study, other important barriers that needed to be noted are, (n= 815) participants 73% feared catching an infection, (n= 249) 22.3% were afraid of causing harm, and (n= 478) 42.8% reported psychological barriers to providing first aid. Similarly, Barnawi et al. [7] found that 178 participants (20.9%) feared disease transmission, and 627 (73.6%) feared harming the injured person. In a study among the general population in the Al-Majma'ah region reported comparable concerns: 28 (4.2%) feared infection, 108 (16.1%) feared causing harm, and 13 (1.9%) cited emotional factors [18]. However, most of their participants (383, 57.1%) were unsure about the main reason preventing CPR in public. It is understandable to fear acting in such situations, as concerns may arise about contracting diseases or unintentionally causing further harm. Thus, further research is needed to identify other barriers as it mentioned in other studies.  

In this study, (n= 810) 72.6% avoided giving first aid due to discomfort with being the center of attention, likely linked to fear or low confidence. Similarly, in India, fear (59.9%) and lack of confidence (17.7%) are key barriers among non-healthcare professionals [21]. Another barrier was discomfort in assisting the opposite sex, with (n= 511) 45.8% agreeing and (n= 336) 30.1% disagreeing. Similarly, in China [22] it was found that women were more willing to assist female victims, while many men hesitated due to concerns about physical contact during CPR. 

Strength & limitations****

One of this paper’s major strengths is identifying some underlying barriers to first aid providing, also mentioning the clear needs of the community and support for first aid education. A notable strength is the use of varied and targeted survey questions to explore the different aspects of hesitation in providing first aid, allowing for a more nuanced understanding of underlying causes. Additionally, the study benefits from a large and diverse sample, including over 1,100 participants from various age groups and regions across Riyadh. On the other hand, some limitations were identified that needed to be mentioned. Because convenience sampling was used, the sample may not fully represent the broader Riyadh population, limiting the generalizability of the findings. Moreover, as a cross-sectional study, the design limits the ability to draw causal inferences between the identified barriers and actual first aid behaviors, and the use of convenience sampling might also introduce some sort of biases. The reliance on self-reported data introduces potential for response and recall bias, such response and recall biases may introduce measurement inaccuracy and influence the reliability of the findings. Another limitation of this study is the potential underrepresentation of older adults, as the use of social media platforms for survey dissemination may have limited participation among less digitally active adult and elderly populations. Lastly, the study focuses only on Riyadh, limiting the ability to understand differences across other regions in Saudi Arabia.

Recommendations****

Future research is encouraged to include detailed educational levels (e.g., diploma, bachelor's, postgraduate) to better understand how academic degrees may influence first aid knowledge and practice. It is also recommended to conduct qualitative studies to gain deeper insight into the psychological and social factors contributing to hesitancy in providing first aid. To strengthen first aid readiness in the community, initiatives should focus on expanding free and certified training programs, especially targeting non-healthcare individuals. Public awareness campaigns and social support systems should be enhanced to address mental and social barriers. Policymakers and health authorities should also assess the effectiveness of current training programs by comparing outcomes before and after implementation. 

## Conclusions

This study explored key psychological, social, and consequential barriers preventing bystanders in Riyadh from providing first aid during emergencies. While many participants expressed confidence and support for first aid, a significant portion hesitated due to fears of causing harm, legal consequences, and social discomfort, particularly when assisting the opposite sex or acting in public. The gap between general awareness and formal certification further highlights the need for structured, accessible training programs. Importantly, among Riyadh participants, misconceptions that first aid is solely the responsibility of healthcare professionals remain. In the context of Riyadh, the findings suggest a potential benefit to integrating certified first aid education into schools and workplaces. 

## Appendices

**Table 4:** Questionnaire Instrument in English

Section	Q#	Question	Response Options
Sociodemographic Characteristics	1.1	Region of Riyadh	☐ North of Riyadh ☐ South of Riyadh ☐ West of Riyadh ☐ East of Riyadh ☐ Central Riyadh ☐ Outside Riyadh
	1.2	Gender	☐ Female ☐ Male
	1.3	Age	☐ 18–20 ☐ 21–25 ☐ 26–30 ☐ 31–35 ☐ 36–40 ☐ 41–45 ☐ 46–50 ☐ 51–55 ☐ 56–60
	1.4	Occupational status	☐ Student ☐ Employed ☐ Employed & Student ☐ Unemployed
	1.5	Do you work in / have knowledge in the health field?	☐ Yes, I have knowledge ☐ Yes, I work in the health field ☐ No knowledge/experience
	1.6	Have you taken a first aid course?	☐ Yes ☐ No
	1.7	Do you have a first aid license?	☐ Yes ☐ No
First Aid Awareness & Preparedness	2.1	Can a first aid provider save a life?	☐ Yes ☐ No
	2.2	Teaching first aid is important for students & employees	☐ Agree ☐ Neutral ☐ Disagree
	2.3	How many times have you provided first aid?	☐ None ☐ Some crises ☐ Most crises ☐ Have not faced a crisis
	2.4	What will you do during crises/emergencies?	☐ Provide first aid ☐ Call an ambulance ☐ Direct others ☐ Provide emotional support ☐ Stand and watch ☐ Get away
Psychological Assessment	3.1	Psychological fear prevents me from providing first aid.	☐ Agree ☐ Neutral ☐ Disagree
	3.2	Stress prevents me from giving first aid.	☐ Agree ☐ Neutral ☐ Disagree
	3.3	The possibility of causing harm to the injured person prevents me from providing first aid.	☐ Agree ☐ Neutral ☐ Disagree
	3.4	My fear of the injured person's reaction prevents me from providing first aid.	☐ Agree ☐ Neutral ☐ Disagree
	3.5	Previous bad experiences prevent me from giving first aid.	☐ Agree ☐ Neutral ☐ Disagree
	3.6	I don’t like being the center of attention, so I don’t give first aid.	☐ Agree ☐ Neutral ☐ Disagree
	3.7	Fear of infection prevents me from giving first aid.	☐ Agree ☐ Neutral ☐ Disagree
	3.8	I have health problems that prevent me from providing first aid.	☐ Agree ☐ Neutral ☐ Disagree
Social Assessment	4.1	I feel uncomfortable providing first aid to people of the opposite sex.	☐ Agree ☐ Neutral ☐ Disagree
	4.2	I'm worried about the legal consequences so I don't provide first aid.	☐ Agree ☐ Neutral ☐ Disagree
	4.3	I cannot predict the reaction of the people around the injured person, so I cannot provide first aid.	☐ Agree ☐ Neutral ☐ Disagree
	4.4	I feel uncomfortable giving first aid to strangers.	☐ Agree ☐ Neutral ☐ Disagree
	4.5	Society's lack of understanding of the importance of first aid prevents me from providing it.	☐ Agree ☐ Neutral ☐ Disagree
	4.6	I don't prefer to help when I'm in another community.	☐ Agree ☐ Neutral ☐ Disagree
	4.7	My lack of knowledge of the laws regarding first aid prevents me from providing it.	☐ Agree ☐ Neutral ☐ Disagree
	4.8	Social pressures prevent me from providing first aid.	☐ Agree ☐ Neutral ☐ Disagree
Consequential Assessment	5.1	Difficulty accessing first aid learning resources prevents me from providing them.	☐ Agree ☐ Neutral ☐ Disagree
	5.2	Not having a first aid license prevents me from providing it.	☐ Agree ☐ Neutral ☐ Disagree
	5.3	I feel that providing first aid is difficult and a big challenge.	☐ Agree ☐ Neutral ☐ Disagree
	5.4	I rely on the people around the injured person, so I do not provide first aid.	☐ Agree ☐ Neutral ☐ Disagree
	5.5	Not being sure that the person needs help prevents me from providing first aid.	☐ Agree ☐ Neutral ☐ Disagree
	5.6	I believe that providing first aid is the job of paramedics and specialists, so I do not provide first aid.	☐ Agree ☐ Neutral ☐ Disagree
	5.7	The environmental and climatic conditions surrounding me prevent me from providing first aid (rainy or stormy weather) or (the injured person is in a place that is difficult to reach, such as a public road)	☐ Agree ☐ Neutral ☐ Disagree
	5.8	Social media prevents me from providing first aid (such as someone around me taking pictures - or my unwillingness to contribute to an event that might be circulated on social media platforms)	☐ Agree ☐ Neutral ☐ Disagree

**Table 5:** Questionnaire Instrument in Arabic

خيارات الإجابة	السؤال	رقم السؤال	القسم
☐ شمال الرياض ☐ جنوب الرياض ☐ غرب الرياض ☐ شرق الرياض ☐ وسط الرياض ☐ خارج الرياض	منطقة السكن في الرياض	1.1	الخصائص الديموغرافية
☐ أنثى ☐ ذكر	الجنس	1.2	
☐ 18–20 ☐ 21–25 ☐ 26–30 ☐ 31–35 ☐ 36–40 ☐ 41–45 ☐ 46–50 ☐ 51–55 ☐ 56–60	العمر	1.3	
☐ طالب ☐ موظف ☐ موظف وطالب ☐ غير موظف	الحالة الوظيفية	1.4	
☐ نعم، لدي معرفة ☐ نعم، أعمل في المجال الصحي ☐ لا، ليس لدي معرفة أو خبرة	هل تعمل أو لديك أي معرفة في المجال الصحي؟	1.5	
☐ نعم ☐ لا	هل حصلت على دورة إسعافات أولية؟	1.6	
☐ نعم ☐ لا	هل لديك رخصة إسعافات أولية؟	1.7	
☐ نعم ☐ لا	هل يمكن لمقدّم الإسعافات الأولية إنقاذ حياة؟	2.1	الوعي والاستعداد للإسعافات الأولية
☐ أوافق ☐ محايد ☐ لا أوافق	إلى أي مدى تتفق أن تعليم الإسعافات الأولية مهم للطلاب والموظفين؟	2.2	
☐ لم أقدم أبداً ☐ في بعض الأزمات ☐ في معظم الأزمات ☐ لم أواجه أو أشهد أزمة	كم مرة قدمت إسعافات أولية في المواقف والأزمات التي واجهتها؟	2.3	
☐ تقديم إسعافات أولية ☐ الاتصال بالإسعاف ☐ توجيه الآخرين للمساعدة ☐ دعم نفسي وعاطفي ☐ الوقوف والمشاهدة ☐ الابتعاد	ماذا ستفعل أثناء الأزمات والطوارئ؟	2.4	
☐ أوافق ☐ محايد ☐ لا أوافق	يمنعني الخوف النفسي من تقديم الإسعافات الأولية.	3.1	التقييم النفسي
☐ أوافق ☐ محايد ☐ لا أوافق	يمنعني التوتر من تقديم الإسعافات الأولية.	3.2	
☐ أوافق ☐ محايد ☐ لا أوافق	يمنعني احتمال التسبب بالضرر للمصاب من تقديم الإسعافات الأولية.	3.3	
☐ أوافق ☐ محايد ☐ لا أوافق	يمنعني خوفي من ردة فعل المصاب من تقديم الإسعافات الأولية.	3.4	
☐ أوافق ☐ محايد ☐ لا أوافق	تمنعني التجارب السيئة السابقة من تقديم الإسعافات الأولية.	3.5	
☐ أوافق ☐ محايد ☐ لا أوافق	لا أحب أن أكون مركز الاهتمام، لذلك لا أقدم الإسعافات الأولية.	3.6	
☐ أوافق ☐ محايد ☐ لا أوافق	يمنعني الخوف من العدوى من تقديم الإسعافات الأولية.	3.7	
☐ أوافق ☐ محايد ☐ لا أوافق	تمنعني المشاكل الصحية من تقديم الإسعافات الأولية.	3.8	
☐ أوافق ☐ محايد ☐ لا أوافق	أشعر بعدم الارتياح عند تقديم الإسعافات الأولية لشخص من الجنس الآخر.	4.1	التقييم الإجتماعي
☐ أوافق ☐ محايد ☐ لا أوافق	أخشى العواقب القانونية، لذلك لا أقدم الإسعافات الأولية.	4.2	
☐ أوافق ☐ محايد ☐ لا أوافق	عدم قدرتي على توقع ردة فعل الآخرين يمنعني من تقديم الإسعافات الأولية.	4.3	
☐ أوافق ☐ محايد ☐ لا أوافق	أشعر بعدم الارتياح عند تقديم الإسعافات الأولية للغرباء.	4.4	
☐ أوافق ☐ محايد ☐ لا أوافق	يمنعني عدم وعي المجتمع بأهمية الإسعافات الأولية من تقديمها.	4.5	
☐ أوافق ☐ محايد ☐ لا أوافق	لا أفضل المساعدة عندما أكون في مجتمع آخر.	4.6	
☐ أوافق ☐ محايد ☐ لا أوافق	يمنعني عدم معرفتي بالقوانين المتعلقة بالإسعافات الأولية من تقديمها.	4.7	
☐ أوافق ☐ محايد ☐ لا أوافق	تمنعني الضغوط الاجتماعية من تقديم الإسعافات الأولية.	4.8	
☐ أوافق ☐ محايد ☐ لا أوافق	تمنعني صعوبة الوصول إلى مصادر تعلم الإسعافات الأولية من تقديمها.	5.1	التقييم المتعلق بالعواقب
☐ أوافق ☐ محايد ☐ لا أوافق	عدم امتلاكي رخصة إسعافات أولية يمنعني من تقديمها.	5.2	
☐ أوافق ☐ محايد ☐ لا أوافق	أشعر أن تقديم الإسعافات الأولية صعب ويمثل تحدياً كبيراً.	5.3	
☐ أوافق ☐ محايد ☐ لا أوافق	أعتمد على الأشخاص حول المصاب، لذلك لا أقدم الإسعافات الأولية.	5.4	
☐ أوافق ☐ محايد ☐ لا أوافق	عدم تأكدي من أن الشخص يحتاج مساعدة يمنعني من تقديم الإسعافات الأولية.	5.5	
☐ أوافق ☐ محايد ☐ لا أوافق	أعتقد أن تقديم الإسعافات الأولية هو عمل المسعفين والمتخصصين.	5.6	
☐ أوافق ☐ محايد ☐ لا أوافق	الظروف البيئية والمناخية المحيطة بي تمنعني من تقديم الإسعافات الأولية (مثل الطقس الماطر أو العاصف) أو (وجود المصاب في مكان يصعب الوصول إليه، مثل الطريق العام).	5.7	
☐ أوافق ☐ محايد ☐ لا أوافق	تمنعني وسائل التواصل الاجتماعي من تقديم الإسعافات الأولية (مثل قيام أحد الموجودين بالتصوير – أو عدم رغبتي في المشاركة في حدث قد يتم تداوله على منصات التواصل الاجتماعي).	5.8	

**Table 6:** Detailed Coding and Statistical Procedures

Variable/scale	Original responses	Recoding/scoring	Purpose/notes
Psychological Barriers (Section 3)	3-point Likert scale (Agree, Neutral, Disagree)	1 = disagree, 2 = neutral, 3 = agree (except for Q3 which was revered)	Based on total scores, barrier levels were categorized as follows: 8-13 = Low perceived barrier, 14-18 = Moderate barrier, and 19-24 - High perceived barrier.
Social Barriers (Section 4)	3-point Likert scale (Agree, Neutral, Disagree)	1 = disagree, 2 = neutral, 3 = agree	Based on total scores, barrier levels were categorized as follows: 8-13 = Low perceived barrier, 14-18 = Moderate barrier, and 19-24 - High perceived barrier.
Consequential Barriers (Section 5)	3-point Likert scale (Agree, Neutral, Disagree)	1 = disagree, 2 = neutral, 3 = agree (except for Q6 which was revered)	Based on total scores, barrier levels were categorized as follows: 8-13 = Low perceived barrier, 14-18 = Moderate barrier, and 19-24 - High perceived barrier.
Software used	—	JMP Pro 18, Microsoft Excel 2021	Statistical analysis and data management
Statistical methods	—	Mean, standard deviation, and variance	Calculated to assess the overall intensity of each barrier type, and results were presented in tables and figures for clarity
